# Supplementary material for: MetaQTL: a package of new computational methods for the meta-analysis of QTL mapping experiments
Source: BMC Bioinformatics. 2007 Feb 8;8:49. doi: 10.1186/1471-2105-8-49 (PMC1808479; doi:10.1186/1471-2105-8-49)
Supplement: Additional File 7 — MetaQTL Package : jar file and tutorial. This Zip archive contains both the MetaQTL JAR file and the files of the tutorial. [file 1471-2105-8-49-S7.zip › org.inra.metaqtl/doc/org/metaqtl/Qtl.QTLCross.html]

Qtl.QTLCross


|  |  |  |
| --- | --- | --- |
| **Overview** \| **Package** \| **Class** \| **Use** \| **Tree** \| **Deprecated** \| **Index** \| **Help** | |  |
| **PREV CLASS**   **NEXT CLASS** | **FRAMES**    **NO FRAMES**     **All Classes** |  |
| SUMMARY: NESTED \| FIELD \| CONSTR \| METHOD | DETAIL: FIELD \| CONSTR \| METHOD |  |


---


## org.metaqtl Class Qtl.QTLCross

```
java.lang.Object
  org.metaqtl.Qtl.QTLCross
```

**All Implemented Interfaces:**: java.io.Serializable

**Enclosing class:**: Qtl

---

``` public class Qtl.QTLCross extends java.lang.Object implements java.io.Serializable ```

This class stores the informations on the cross
design used to detect the QTL.

**See Also:**: Serialized Form

---

| **Field Summary** | |
| --- | --- |
| `int` | `gen`             The number of generations. |
| `java.lang.String` | `name`             The name of the cross design. |
| `int` | `size`             The number of individuals. |
| `int` | `type`             The type of the cross. |


**Constructor Summary**

`Qtl.QTLCross()`


| **Method Summary** | |
| --- | --- |
| `Qtl.QTLCross` | `getCopy()` |

**Methods inherited from class java.lang.Object**

`clone, equals, finalize, getClass, hashCode, notify, notifyAll, toString, wait, wait, wait`

**Field Detail**

### name

```
public java.lang.String name
```

:   The name of the cross design.

---


### type

```
public int type
```

:   The type of the cross.

---


### gen

```
public int gen
```

:   The number of generations.

---


### size

```
public int size
```

:   The number of individuals.


**Constructor Detail**

### Qtl.QTLCross

```
public Qtl.QTLCross()
```


**Method Detail**

### getCopy

```
public Qtl.QTLCross getCopy()
```

:   **Returns:**


---


|  |  |  |
| --- | --- | --- |
| **Overview** \| **Package** \| **Class** \| **Use** \| **Tree** \| **Deprecated** \| **Index** \| **Help** | |  |
| **PREV CLASS**   **NEXT CLASS** | **FRAMES**    **NO FRAMES**     **All Classes** |  |
| SUMMARY: NESTED \| FIELD \| CONSTR \| METHOD | DETAIL: FIELD \| CONSTR \| METHOD |  |


---
